# Notes and Comments

**Published:** 1922-11

**Authors:** 


THE
### HOSPITAL and HEALTH
REVIEW

### SURMOUNTING THE HOSPITAL CRISIS.

IT will be remembered that, in the report of Lord Cave's Committee on the voluntary hospitals, it was shown that, of the 672 hospitals in Great Britain whose accounts had been examined, 378 had deficits in 1920, amounting in all to rather more than £1,000,000; it was further considered that the total deficits for 1921 could hardly be less than in 1920. Lord Cave's Committee suggested that State funds should make good the entire deficit for 1921, but succeeded in obtaining only £500,000 from the Treasury, of which grant £166,000 was distributed last year in London and £95,000 in the provinces apart from London. In hospital circles there has been a great deal of speculation as to what the figures for last year would disclose of the financial stability of hospitals, and whether the outlook would be worse or better than in the year reviewed by Lord Cave's Committee. We can now examine the situation with the help of the very able report on provincial and Scottish hospitals made by Sir Napier Burnett at the request of the Joint Council of the Order of St. John and the British Red Cross Society. As did the Cave Committee, Sir Napier has examined the statistics of practically all the hospitals of importance in Great Britain, apart from London; he finds that, taking the hospitals as a whole, there is a net deficit of £420,515. We are able, by means of tabulated figures specially prepared for this REVIEW, to add to the information provided by Sir Napier Burnett. In London, during 1921, 62 hospitals had deficits amounting to £225,000, and 47 hospitals had surpluses amounting to £118,000. Sir Napier Burnett thinks he sees a rift in the clouds, and he bases his optimism on the fact that in 1920 only 44 per cent. of the hospitals surveyed were able to show a financial surplus on the year's working, whereas in 1921, despite the unprecedented extent of unemployment and financial depression, the number of hospitals that were able to make ends meet increased to 51 per cent.

We fear that we cannot place such a bright interpretation upon these figures as does Sir Napier. His statistics and ours together disclose that the net deficiency in the accounts of all the hospitals in Great Britain for 1921 amounted to £527,000. Lord Cave's report showed for 1920 a surplus for 378 hospitals of £427,000 and a deficiency for 294 hospitals of £1,038,000, or a net deficiency of £611,000. In addition, it must be remembered that during 1921 there was distributed, on account of the State's temporary subsidy, the sum of £261,000. The position is therefore that the hospitals, as a whole, did not do as well in 1921 as in 1920. Taking by themselves the lame ducks, or hospitals with an excess of expenditure over income, and combining Sir Napier Burnett's figures with our own, the total deficit for all the hospitals of Great Britain was £929,037, and presumably would have been, but for the State grant, £1,190,000. Lord Cave's Committee's estimate, therefore, turns out to be on the low side.

We would rather base any feeling of congratulation upon the fact that the hospitals have not, as a whole, become much more deeply involved in debt, but have been given breathing space while putting their house in order. New sources of income have been opened up. The smaller giver is now for the first time organised. The principle of payments by patients is definitely accepted. Sir Napier Burnett thinks that the payment system should be extended to include people above the present hospital income limit, a class notoriously badly catered for. In other words, the American system of hospitals for rich and poor is advocated. We have no doubt that this will come, but our hospitals, as they stand, cannot be used in this way without alteration or enlargement. This involves capital expenditure which cannot be entertained at the present time. But the system must be prepared for by study and discussion. It will, for one thing, be popular with the honorary medical staffs of hospitals and a means of settling the vexed question of payment without destroying all that voluntary medical service to the needy has always stood for.

We agree with Sir Napier as to the deadly dullness of the average hospital report and the waste of excellent journalistic material which is hardly ever employed to gain and hold the sympathy and support of the public. To quote Sir Napier's words, "If hospitals will realise their opportunities in this matter and seek, not only through the columns of the Press, to instruct the public in the great work in which they are engaged, namely, treating the sick, investigating the nature and cause of disease, and thus advancing preventive medicine, and also in the training of doctors and nurses, who are to serve in the homes of the people, then the public would take renewed interest in the 'saving of their hospitals.'" We commend these words to appeal secretaries. Properly applied and circulated, they should bring in many thousands to our voluntary hospitals.

---

### THE OUTSIDE OF THE TIN.

THE tragedy of Lochmaree has directed attention to the processes connected with the canning of food. In a recent article the "Lancet" refers to the physical alteration of the outside of the tin which usually—though not always—accompanies putrefaction of its contents. The canning of foods and the processes of soldering were thoroughly dealt with in its columns some years ago by Dr. F. W. Alexander, medical officer of health for Poplar. From his large experience in following legal proceedings relating to defective canned foods he brought to light several interesting facts—*e.g.*, that the "blow-hole" is not really necessary, but that firms supplying canned food make the hole during the first or low-heat stages of the bathing and steaming processes to allow air to escape, so that when the cooking and sterilising are complete, and the cans have cooled down, the ends of the can become concave. This is done, Dr. Alexander was assured, to satisfy the public who like to see concave ends when purchasing.

The question of how long foods hermetically sealed will keep was raised during the hearing of the summonses, and whether the heat of a shop would not affect the contents of a can. Dr. Brown, medical officer of health for Bacup, states: "Fortunately, having had canned foods, including meats, soups, rabbits, oysters, etc., of from 20 to 30 years old, I found that the tins, though rusty outside, were perfectly good inside; none were blown." One of the experts for the prosecution told Dr. Alexander that his firm had canned meat quite good which had been through the Crimean War. Another expert stated that one plan adopted by his firm to test whether the sterilising process had been properly carried out and whether the contents of cans were good before sending them out was to place them in a heated room or chamber in order to see if the cans became blown. Dr. Alexander suggested that no can should have more than one soldering-point, and that this point should be either at the top or the bottom of the can, in such a position that it is not liable to be covered by a label; before the can leaves the factory a metallic plate with the name of the firm and the date of sending out should be soldered over the soldering-point. Imported canned foods should have the names of the importers printed on the label, and such persons should be made answerable for the same requirements.

---

### BOTULISM.

DR. MEYER, who is in charge of the George Williams Hooper Foundation for Medical Research at the University of California Medical School, San Francisco, has been studying botulism for over three years. In an interview with a representative of the HOSPITAL AND HEALTH REVIEW he stated that the article in our last issue was extremely accurate and well balanced, but that in certain respects the figures quoted, which were obtained from British sources, could be brought more up to date. Since 1899 the United States have had 110 outbreaks, involving approximately 395 persons, of whom 249 have died. In Germany there have been 68 recorded outbreaks, of which 67 were caused by preserved meat, chiefly pork. The outbreak at Lochmaree was the first known in Great Britain. Only one outbreak has been recorded in France, one in Switzerland, two in Belgium and two in Denmark. In view of these figures Dr. Meyer holds that too much has been made of botulism, which "as a cause of death is utterly negligible. If it were not for the dramatic character of the outbreaks, it would have escaped public notice." The prominence given to it may damage well-conducted industries and make the public unduly afraid of harmless forms of food. He considers that the risks in the United States, although infinitesimal as compared with the population, are far greater than those in this country. No less than 70 per cent. of the outbreaks in America have been due to vegetables, mostly those preserved at home. Preserving is far more popular on the other side of the Atlantic than on this, and the main risk occurs when the housekeeper hunts bargains in the open market. "Fresh and sound raw materials practically exclude botulism."

In the majority of cases, both in Europe and America, botulism has developed in preserved food on account of under-sterilisation. This is due to the fact that original studies of bacteriologists suggested the relatively low heat resistance of the spores. Later investigation at the University of California has proved that the spores may survive boiling for 5½ hours. In many food pastes it takes a long time for the heat to reach the centre of the mass.

Dr. Meyer's studies have revealed the interesting fact that the organism is a common anærobe of the soil, and is found quite frequently in the virgin soil of the mountains. In soil products, whether vegetable, fruit or animal, foods may carry these spores anywhere and at any time. Dr. Meyer insisted: "In America we have to live with the bacillus botulinus," and this has been appreciated by the canning interests, which are making every possible effort to prepare and preserve food free from the organism and the poison. "Mere ingestion of the spores without the poison is harmless. Only when the organism has developed its poison in the course of its growth is it harmful. It should be emphasised, however, that botulism in preserved food is very exceptional. Of 19,000,000 glass containers of olives packed in 1919 and 1920 and examined, only 15 were found to contain any trace of the poison." Contrary to experience in this country, in the majority of cases signs of spoilage were obvious. In blown tins the product emanates an odour of rancid butter, and as a result the poisoned cans can be quickly eliminated.

### EX-SERVICE MEN IN ASYLUMS.

In its eagerness for the sensational and the scandalous the daily press has made an unfounded attack on the Ministry of Pensions. Statements have appeared to the effect that all ex-Service men in asylums who are at present being paid for by the Ministry as "Service patients" will no longer be paid for after Sept. 30 and will become pauper patients. The Ministry has issued a statement pointing out that the State is liable to pay compensation and to provide medical treatment for all men disabled by war service, including those who, though they before enlistment suffered from some complaint, were, nevertheless, and remain, worsened in consequence of their service. A certain number of men enlisted already with disabilities of various kinds, including mental affection, and were discharged without any worsening of their disabilities. For these men (who, under the Royal Pension Warrants in force before the Ministry of Pensions was instituted, were simply discharged from the Service without title to receive anything) special provision was made, of a temporary compassionate nature, by the first Pensions Ministry Warrant of 1917, by which they were given a lump sum gratuity or a small weekly allowance for a time, and the Ministry of Pensions was empowered to give them treatment for the period of the war and one year after. The fact that the provision of medical treatment and certain allowances was temporary only has been known from the first. Moreover, the decision of the Army doctors and the Ministry doctors after examination of the men, that their war service was not responsible for their disablement, has been for the last five years subject to the right of appeal to an independent body—the Pensions Appeal Tribunal.

Appeal has been made on behalf of many of the men to these tribunals, and their cases gone into with results either favourable to the men or confirmatory of the Ministry's original decision. Further, in order to prevent the possibility of any injustice being done Mr. Macpherson has taken special steps to bring to the notice of the relatives that there is this right of appeal. If the decision in any of these cases is favourable to the man he will retain his title to be a Service patient in the asylum.

The Minister of Pensions remains responsible for the maintenance and treatment of between 5,000 and 6,000 ex-Service patients, who are in asylums either temporarily or permanently, and whose mental condition is due to war service. There has been no fresh decision or Order by the Ministry. What has happened with regard to the men whose mental condition is not in any way due to war service is only the carrying out of the direction of the Royal Pension Warrants of the last five years—a direction approved by Parliament itself.

### RAPID FALL OF THE TUBERCULOSIS DEATH-RATE IN NEW YORK.

In the U.S.A. and in New York City in particular the tuberculosis death-rate appears to be tumbling down at an amazing rate, and in a recent paper on this subject in the "American Review of Tuberculosis" for June, 1922, Dr. Haven Emerson has calculated that if this fall is made continuous "we can foresee the eradication of tuberculosis as a disease of importance within the next 20 years in this country." Did he not support his argument by reliable statistics, his claim would suggest exaggerated optimism. But there is no doubt that in the past 50 years the tuberculosis death-rate in New York City has fallen 77·9 per cent.; in the past 11 years 51 per cent.; and in the past calendar year of 1921, it has actually fallen 18·1 per cent. The forces which have contributed to this achievement are many and varied; some represent deliberate frontal attacks, such as early diagnosis and treatment, while others, such as the state of general prosperity, have played an accidental part. One of the most remarkable of the direct attacks on tuberculosis is the compulsory pasteurisation in New York of milk and milk products, except such as come from herds or cows proved to be free from tuberculosis. In 1914, when this reform was introduced, the rate of non-pulmonary tuberculosis was 27 per 100,000. In 1921 it was only 14 per 100,000. In the period 1912-1913, 64 per cent. of a series of 100 cases of tuberculous glands were traced to bovine infection, and in 1917, only 16 per cent. of such cases were traced to infection with tubercle bacilli of the bovine type. It is not clear whether or not adult pulmonary tuberculosis has been affected by 98 per cent. of the City's milk being pasteurised, but it is evident that the reduction of milk-borne, lymphatic tuberculosis of children is an accomplishment of great and lasting importance. Another paper on the same subject, in the "Bulletin of the New York Tuberculosis Association" for May and June, shows that between 1910 and 1920 the tuberculosis mortality has been reduced more than twice as fast as the general mortality in New York, the reduction for the latter being only 19 per cent., as compared with 40 per cent. for the tuberculosis mortality. The largest reduction in any one age group was among children under the age of one year, the reduction being 46 per cent. It is, of course, difficult, and to a certain extent impossible, to apportion the praise for these achievements, but probably they are largely due to the Andvord-Römer teaching that pulmonary tuberculosis in adult life is contracted in infancy or early childhood. Working on this hypothesis, the health authorities in New York and Chicago have focussed attention on preventing young children from being in close contact with open cases of pulmonary tuberculosis, and the fruits of this campaign are already in evidence. What is not yet evident is the saving of lives which would probably been lost some 20 to 40 years hence, had not the U.S.A. authorities secured isolation of children from infectious cases.

---

### EDUCATION AT THE COST OF HEALTH.

*MENS sana in corpore sano* is a maxim sometimes given only lip-service, and certain teachers are apt to think only in terms of examinations, scholarships and other prizes. A useful corrective to this tendency is to be found in a paper recently published by Captain N. C. Hertzberg and Dr. Carl Schiötz, Chief of the School Medical Service in Norway. They have recorded the chest, height and weight measurements of 70 boys between the ages of 12 and 16 examined from time to time between Jan., 1920, and Jan., 1921. Other investigations have shown that in adults, and children below the school age, there are seasonal changes in the vitality, following a very definite course, as shown by weekly gains or losses of weight. This vitality curve, when it is uninfluenced by school work, runs practically horizontal from Dec. to May—the period of lowest vitality. The curve rises from May to Sept. and then uniformly falls to Dec. Vital statistics show the same tendencies; the highest death-rate is in Feb., the lowest in Sept. In other words, the first half of the year is marked by a comparatively high mortality and low vitality, as judged by the body weight. The vitality curve drawn by the writers shows that school terms affect the physiological curve not a little, and the Christmas and Easter holidays show a rise in vitality which is peculiar to school children. The writers show a canny appreciation of the fact that reforms of the school curriculum dictated only by questions of physical health are likely to be looked at askance by the orthodox pedagogue, but they take their courage in both hands and suggest fairly sweeping reforms. At the present time the duration of the autumn term is only 103 or 104 days, whereas the spring term lasts from Jan. 9 to June 30 (one week's holiday at Easter) with 132 to 133 school days. In other words the season of lowest vitality is that into which the greatest number of school days is crammed. The writers recommend longer Easter holidays and a summer holiday beginning from the first instead of the last day in June. The autumn term should begin earlier and should include 119 to 120 school days, and the spring term only 99 to 100 school days. The conditions are, of course, not exactly parallel in Norway and this country, but both countries are subject to practically the same seasonal changes, and are liable to suffer from the educational fanatic to whom a full curriculum is everything, and the health of the scholar is of secondary importance.

---

### THE NEW ELIXIR OF LIFE.

WRITERS who undertake to enlighten the public concerning its health may be classified according as they are, or are not, legally qualified to sign a death certificate. The former are a well-meaning class, but they are often rather dull. They are apt to linger over facts and the logical deductions therefrom, whereas the non-medical writer, unhampered by a conventional scientific training, rapidly flits on to what is really interesting, deliciously thrilling and imagination-inspiring. He runs, and even flies, where the trained scientist scarce dare walk. Recently, in the daily press, a notice appeared with the heading "New Elixir of Life." What could be more titillating to the senses, more suggestive of a happy excursion into the Arabian Nights? The writer, who is evidently not a doctor, states that enormous interest has been aroused in medical circles by the discovery of a wonderful serum product "which will prolong life to 150 years." But why only 150 years? Surely the serologist or bacteriologist who can prolong life to 150 years could also prolong it to, say, 1,500 years or more. There is something so disappointingly finite about a mere trifle of only 150 years. And how has the writer come to fix on just this term of years, and not 140 or 160? According to "an eminent lung physician, who shares Dr. Spahlinger's secret, the bacteriologist's idea is to inject a serum which so revivifies the surrounding tissues that it infects them with the rejuvenescence." It is doubtful if we shall find much time to spare even when our span of life is extended to 150 years. Methuselah had certainly no time to throw away. He was, on the contrary, frightfully rushed. In an article entitled "The Art of Procuring Pleasant Dreams," Benjamin Franklin published in September, 1786, in the *Columbian Magazine*, of Philadelphia, an instructive account of Methuselah. When he had lived 500 years, an angel said to him, "Arise, Methuselah, and build thee an house, for thou shalt live yet 500 years longer." But he answered, "If I am to live but 500 years longer, it is not worth while to build me an house . . . . I will sleep in the air as I have been used to do." The elixir of life, like the philosopher's stone, has intrigued and held the imaginations of philosophers of countless generations, but without wishing to be fulsome in our praise, we would suggest that none of them has got nearer to the solution of the problem than Dr. Spahlinger's journalistic impresario. The cynic might object that a claim, the testing of which must be deferred a century or so, is easier to make than to disprove, but the Spahlinger elixir has immediate as well as remote effects. "The ferment injections simply make me feel as strong as a lion." It also "gives the look of youth to the face as well as imparting the throb and vigour to the blood and body. Its effect is to smooth out all the lines and creases and wrinkles of age. Death seems ridiculously impossible." Samson redivivus, in short. Samson, also, it may be remembered, knew how to make good play with the jawbone of an ass.

### A NEW DISEASE.

THE comprehensive report on encephalitis lethargica, written by Dr. Allan C. Parsons, has just been published. It contains over 300 pages, analysing the cases of this new disease that have been notified since Jan. 1, 1919, with a full bibliography, and contributions by Dr. MacNalty and Dr. Perdrau, pathologist at Lambeth Infirmary, summarising their scientific investigations into the disease.

The first intelligence of this strange malady was received from Vienna in 1917. A few cases were reported from Sheffield and London in the spring of 1918. Very promptly an inquiry was made by the Local Government Board, and it is satisfactory to know that the provisional conclusions then arrived at are confirmed by the experience of over two years. This disease is not connected with botulism or with influenza, and certainly not with the sleeping sickness of the tropics, which is due to the bite of a fly; the lay press, by using the popular phrases "sleeping sickness" and "sleepy sickness," has misled the public. It is a new clinical concept. The manifestations of lethargy, followed by stupor, have impressed the public imagination. The inquiry shows that the disease is apparently due to a germ which finds its point of entry through the nose, and this affects the brain. The virus was discovered two years ago, and was transferred successfully to a patas monkey. As the result of this and other investigations, medical knowledge on the subject has been considerably extended, more in a negative direction than positively. The way in which the disease is spread is still obscure. It has not yet proved possible to determine those persons who are the unconscious reservoirs of the virus. Nevertheless, the inquiry makes it possible to have some idea of the way in which the disease should be treated, and to distinguish between cases that will probably prove fatal and others. The mortality rate is well over 40 per cent., but the number of cases this year shows a heavy decline, and Sir George Newman states in the report that there is good reason to think that the cases are becoming milder.

The report is a storehouse of facts and figures on this subject. It proves the wisdom of those who insisted that the disease should be made notifiable. This was done over a year before similar measures were adopted in any foreign country, and therefore in ascertaining the causes of this mysterious new disease British medical research is leading the way.

### HOLIDAY CENTRES AND EPIDEMICS.

WE lately drew attention to the problems arising from London children who when on holiday develop the symptoms of infectious disease that they have brought from town. London is not the only object of local censure in this matter. During the recent holiday season infectious disease has made its appearance among visitors at several places, and perhaps in Wales has been indirectly useful in so doing. Dr. Hugh Jones, medical officer to the Dolgelley Council, has been notified of a case of scarlet fever and a case of measles, the patients being a visitor's child from Stockport and a Manchester Sunday School child with a seaside party. Dr. Jones used these cases to reinforce the demand that he has made, he says, for twenty-five years, the demand for an isolation hospital. Merioneth County Council, he added, deserved the severest censure for its apathy in permitting the county to be without a hospital of this kind. It is certainly clear that local precautions cannot suffice where there is an influx of visitors, and should a makeshift isolation prove insufficient, as is always possible, an epidemic would cure the neglectful resort of visitors for a long time to come. The local authorities then would have no defence from the enraged caterers who had lost their income.

How widespread the dangers are, and the importance of an isolation hospital, simultaneously appear from East Anglia. Dr. Barnes, medical officer of health to the Hartismere Rural District Council, has reported an outbreak of scarlet fever among children brought to Mellis from the Walworth district of London by the Children's Holiday Fund. To a small cottage with only two bedrooms, occupied already by a man, his wife and grown-up daughter, six of these children were transferred. Some were promptly down with scarlet fever, but there was an isolation for them to go to, and the rest were segregated. The children stated that there had been an outbreak of scarlet fever and diphtheria in the district from which they came, and Dr. Barnes supposed that they had brought the infection in their clothing. The house where they were boarded was already overcrowded, and its occupants should not have been allowed to receive so many children. The London authorities should also have been more careful. He had written to them, but received no reply. Responsibility was divided between the children's host, the person who arranged for their reception at Mellis, and the dispatching authority in London. A protest to the Children's Holiday Fund was suggested, and we agree with Dr. Barnes that if the London authorities would communicate in advance with the medical officer of the districts to which the children were to be sent, such cases should not occur. This was, we believe, the custom before the war. It should certainly be revived, and the decision to apply to the Children's Holiday Fund for the cost of treating the children will probably secure a return to it.

### THE BOLSHEVIST BLIGHT.

THE eminent Russian economist, Prof. P. Sorokin, one of the first group of intellectuals to be expelled from Russia, has read a report in Berlin before an assembly of journalists, professors and others on the state of that country, which is summarised in the "Times." The facts which relate to public health are, like the rest, appalling. The population in 1914 was equal to 160-176 millions; it had decreased to 129 millions in 1921. Not counting the territories which fell away after the revolution, the loss of population within the boundaries of the Soviet Republic itself amounts to 21 millions. Parallel with this decline the biological and intellectual standard has deteriorated. The war, and principally the civil war, killed off the strongest elements of the population. The ravages caused by the Bolshevist administration in the ranks of the intellectuals is very great. The death-rate amongst them has been thrice that amongst the rest of the population. The elements spared by the war were mostly inferior in morals and physique, and they, too, have deteriorated as a consequence of famine, cold and disease. The results are shown in the smaller growth of the children, the decrease of weight of newly-born babies, the weakening of their vitality, and the increase of tuberculosis, scurvy, and venereal disease.

The Russian revolution has brought no amelioration in the lot of the people. Merely a shuffling of the social structure has occurred, because social inequality is more in evidence now than even in pre-revolutionary times, and far greater than in any capitalist country. The place of the old bourgeoisie and aristocracy was taken in the first part of the revolution by representatives of what was the lower class. But now these are giving place to "specialists," who are members of the former bourgeoisie and even aristocracy. The present system combines all the defects of Tsarism and capitalism with none of their virtues. What an object lesson for the preachers of social revolution in this country! Not even the equality which they demand so loudly, has been achieved, and the lot of those whom they are pleased to confine the term "workers" is infinitely much worse than under the old form of government with all its defects.

### THE DANGERS OF HAIR DYES.

AN illustration of the dangers of hair dyes used by barbers is given in a recent issue of the "Long Island Medical Journal," by Dr. W. Gilman Thompson. A woman of 55, while travelling in England, suffered from a dermatitis behind the ears, over the neck and extended over the shoulders. A well-known English physician, whom she consulted, treated her for gout, but periodically her condition became worse and the eruption spread. She consulted another physician, who said that the gout theory was all wrong. His view was that she was a society woman and much overworked, and needed rest and building up. His treatment was diametrically opposite to that of his colleague. In the meantime she consulted various dermatologists, who treated her by ointments. Nevertheless the eruption recurred every few weeks and spread so that the lady could not wear evening dress, and had to give up all social functions.

She returned to the United States, and there was treated at a well-known spa, where she was given a series of baths, which much reduced her strength and extended the trouble over a considerable portion of her body. Another American physician declared that she had a weak heart, and treated her for this without result, except that she became more depressed and seriously ill. Finally Dr. Thompson was called in. He noticed that her hair was of a much more brilliant Titian red than formerly. This gave him the clue to the cause of the trouble. He accused the patient of using hair dye. She denied the charge with much vehemence, but after an altercation two bottles were produced. When their contents were tested it was discovered that the dye contained an aniline dye so poisonous that its manufacture had resulted in the death of several employees in Germany and had been prohibited there. After giving up the use of the dye, the patient promptly recovered and has been in robust health ever since, but the Titian red has been supplanted by a "grandmotherly grey."

### JENNER ANTICIPATED.

IN the "Times" Mr. Alan R. Goldie calls attention to a fact not generally known. In the graveyard of the ancient Dorset church of Worth Matravers, among the Purbeck Hills, there is a headstone bearing the following inscription: "Sacred to the memory of Benjn. Jesty (of Downshay), who departed this life April 16th, 1816; aged 79 years. He was born at Yetminster in this county, and was an upright honest man; particularly noted for having been the first person (known) that introduced the Cow Pox by inoculation, and who from his great strength of mind made the experiment from the (cow) on his wife and two sons in the year 1774." The anticipation, in some form or other, of a great discovery, whether in medicine or in other sciences, is the rule. Harvey's discovery of the circulation of the blood and Lister's discovery of antiseptics were foreshadowed. Indeed, it is for this reason that controversies as to priority are so common; but it is rightly held that to the man who forces a discovery on an unwilling world the great credit is due. But let us also yield all honour to the humble Dorset farmer who successfully vaccinated 22 years before Jenner. It is opportune to recall the history of vaccination at a time when ignorance and prejudice have led to widespread neglect of the practice. It has been estimated that 60 per cent. of babies now are not vaccinated. Hence the concern with which medical officers of health view the recent outbreaks of smallpox. In one district of Derbyshire over 30 cases have occurred, mostly in unvaccinated children. Prompt isolation and the vaccination of "contacts" have so far saved us from an epidemic, but will they always ?

---

### MOTOR ACCIDENTS.

THE introduction of motor transport has brought one great evil—an increase of vehicle accidents. The number of persons killed in the streets of London, mostly by motor vehicles, has averaged as many as five per week. How this terrible toll of life can be reduced is a pressing subject for inquiry. Nearly a year ago the Ministry of Transport began an investigation into the cause of road accidents, which should prove valuable. The following is an analysis of the causes of 94 accidents in the present year. It will be noticed how large is the percentage due to negligent driving :—

| Cause of Accident. | No. of Accidents. | No. Killed. | No. Injured. |
|---|---|---|---|
| (a) Negligent driving .. | 55 (58·5%) | 35 (46·6%) | 90 (60·8%) |
| Faulty judgment .. | 6 | 17 | 5 |
| Inexperienced driver | 1 | 1 | — |
| (b) Insufficient lighting.. | 1 | — | 2 |
| Faulty steering gear | 5 | 2 | 12 |
| Burst tyre.......... | 1 | 1 | 4 |
| Mechanical defect .. | 1 | — | 1 |
| Faulty brakes ...... | 4 | 4 | 9 |
| Back axle failure .. | 1 | — | 2 |
| Changing gear on steam roller going down gradient .. | 1 | — | 2 |
| Skidding .......... | 4 | 2 | 1 |
| (c) Pedestrian at fault .. | 5 | 5 | 5 |
| Pedal cyclist at fault | 1 | — | 1 |
| Alighting from moving bus .......... | 1 | — | 1 |
| Horse unattended took fright ...... | 2 | 1 | 2 |
| (d) Unknown .......... | 5 | 7 | 2 |
| Total .......... | 94 | 75 | 139 |

### THE FUMIGATION OF SHIPS.

LAST month we referred to the two deaths at Southampton from hydrocyanic acid used in the fumigation of ships. During the year ending June 30, 1921, about 10,000 vessels have been fumigated at the quarantine stations of the United States by various methods, the majority by hydrocyanic acid. Some 2,000 vessels have been thus fumigated in New York during the last 14 months. Fatal accidents have occurred, especially during the time when this fumigation was in the hands of the local authorities, and before the work was taken over by the public health service of the Federal Government. Experimental stations have now been set up at Newport News, Virginia. An exhaustive series of experiments is being carried out on a cargo vessel placed at the disposal of the authorities by the United States Shipping Board. In the opinion of the experts, the dangers involved in the use of hydrocyanic acid are no greater than those in any other dangerous occupation. No opportunity should be lost in this country of investigating the process, with a view to discovering whether any new safeguards are needed.

### LIQUID EGGS.

THERE have been comments in certain quarters as to the dangers of liquid eggs. Last year approximately £7,000,000 worth of eggs from China were imported into this country. It is satisfactory that great improvements have been made in this trade during the past few months, in consequence of the demands of public health authorities. Port sanitary authorities for many years have been releasing only certain classes of preserved egg yolk for industrial purposes, and regulations have to be fulfilled before they can be released for food purposes. The liquid egg made from this material now contains less boric acid, and its use in confectionery has been limited to amounts so small as to be innocuous. The practice of selling liquid eggs in 12 oz. packets to the public was discontinued by the trade some years ago, in view of objections raised by public health authorities. There has recently been some misunderstanding on these points among sanitary inspectors and others. The information above represents the official view up to the present month.

---

### THE FATHERS' COMMITTEE.

THE North Islington Infant Welfare Centre has formed a Fathers' Committee, and this is said to have been so successful that the hon. secretary, Mr. R. W. Rollason, is endeavouring to arrange for similar committees to be started at every London centre. Members of the committee meet on the third Thursday in each month to discuss questions of the milk supply and other issues important to children. When a father refuses to let his child come to the centre for dental treatment or other medical care, a member of the Fathers' Committee usually visits the recalcitrant parent and endeavours to persuade him of the value of infant welfare. It is found that a man can be more successful in dealing with the prejudice and ignorance of fathers than members of the women's committees. Too often in the past all consideration has been given to the mother and none to the father. This new development is useful as an indication that the father's point of view must be considered.

---

### EUTHANASIA.

EUTHANASIA is a topic which constantly recurs in the daily press. The proposal of a Labour member of the Bath City Council to ask the Minister of Health to bring before Parliament a Bill to empower a medical tribunal to effect the more speedy and peaceful end of sufferers from cancer, has produced a flood of controversy. The objection to the proposal is the moral one, which is so strong as to keep it outside the field of practical politics. The medical profession will not assume the duty of ending life. The reason for the proposal—to put an end to suffering—is not so strong as it looks. Suffering can be relieved by large doses of morphia and other narcotics. When a patient is the victim of a painful disease, and the end is already in view, these drugs may be given to any extent.

### LABOUR HOSPITALS IN BELGIUM.

A LARGE hospital has lately been opened in Seraing (Liege) by the Federation of Socialist Mutual Benefit Societies. Seraing is the site of the main John Cockerill iron works, founded by an Englishman, whose products are world-famous. Throughout all this district, the "Black Country" of Belgium, the Labour party is very powerful, and is distinguished, as in other parts of Belgium, by its practical work for the betterment of its members. The new hospital stands in its own grounds, and is equipped with the latest conveniences. It has been made possible by the federalisation of mutual benefit societies, which, 30 years ago, were divided into such small groups that no great action could be undertaken. To-day more than 364,000 families are affiliated, whereas in 1920 there were only 283,000. Special women's groups contain 8,500 members, while other women are grouped with the men. Owing to the wide character of the work undertaken, over half-a-million persons have benefited from the Mutual Societies. The benefits paid vary from 3 to 6 francs per day, according to the district, for the first five weeks, and 2 to 5 francs for re-insurance. Sick benefit is not less than 5 francs, or for slight infirmity 3 francs, and the same for re-insurance. Some federations give maternity benefits and help to widows and orphans. These departments are run by women.

The co-operative "mutualists," as the members are called, have also a hotel, where a thousand people stayed during 1921. The object is to provide the poorer members in the height of the season with a holiday *pension* at reasonable rates, and, during the rest of the year, to serve as a convalescent home.

"Mutualists" in Brussels have also put forth great efforts. A clinic for members was opened in May, 1921, where 30,000 consultations were given in six months, the total cost being 86,000 francs. The accommodation is not sufficient, and the rent is very high, so the societies decided to acquire a large building in the Rue Philippe de Champagne; this is now being altered to fulfil its new purpose, and to allow for extensions to the medical service. The funds for the purchase of the building had to be borrowed from the central co-operative societies.

Where no "mutual" hospital existed, members were often in difficulties regarding the cost of surgical operations. From January 1, 1923, therefore, a new insurance called "operation assurance" will be instituted, which will provide 75 per cent. of the cost of an operation to a maximum of 700 francs. Members will be called upon to pay 6 francs a head yearly (maximum 24 francs per family) for this benefit, which will be extended also to members of their family living under the same roof and dependent upon them.

---

### THE POOR LAW PATIENT'S RIGHT TO DISCHARGE.

THE Kingston coroner lately criticised the law regulating Poor Law Hospitals because a patient, who under it could not be prevented from taking his own discharge, had died, the next day, in his own home. It appeared at the inquest that the patient, who was suffering from skin disease, insisted on taking his discharge after four days, and that his death resulted from septicæmia. He had made his condition worse by scratching his sores. Dr. Davies, chief medical officer of the Kingston Infirmary, explained that both doctors and nurses had tried to persuade the patient to remain in hospital, but that they had no power to detain him against his will. The coroner said that if the hospital authorities had detained the patient, they would have been liable to prosecution, but, despite our fondness for talking of liberty, it would have been better for the patient if he had been chained to his bed. It was sometimes against a man's own interest that he should have liberty. Precisely, but liberty consists in allowing a man any action by which other people's is not invaded, and therefore the coroner does not believe in liberty at all. In this matter the law of 1874, under which Poor Law Hospitals are regulated, is humaner and more statesmanlike. For it is more important that a patient's traditional rights should be respected than that their exercise should occasionally be abused. In fact, to rescind the right for fear of such abuses would achieve a "reform" that would be the worst abuse of all. It would startle Charles Dickens to find the Poor Law of 1874 (four years after his death) criticised because it allows too much liberty to poor law patients. Meantime it is natural for the staff of Kingston Infirmary to regret the failure of its persuasive powers.

---

### DOCTORS AND THE COUNTY HOSPITALS.

SIR NAPIER BURNETT has opened the Insch and District War Memorial Cottage Hospital, Aberdeenshire. Six-sevenths of the cost, £7,500, has been raised, and in addition an endowment fund of £3,000 has been collected. The new hospital is intended to help the opener's policy of decentralisation by relieving the demand on the Aberdeen Infirmary. Insch Hospital accommodates twelve patients. An interesting point in his speech is that the six Scots hospitals with medical schools, of which three are in Glasgow alone, treat more than half of the hospital patients of Scotland. Sir Napier also said that, while the population of Scotland had increased by 2·9 per cent. in the past ten years, the number of patients admitted to the Edinburgh Royal Infirmary had increased by more than 9 per cent. Ninety per cent. of hospital beds were daily occupied in the large city hospitals and 60 per cent. in the cottage hospitals. In other words, there were empty beds in the latter, and the big hospitals tended to be overworked. Yet in the county there are only 0·5 beds per 1,000 of population. Why, then, are there empty beds in the county hospitals? Because many county people occupy beds in the city infirmary. The county hospitals should be more economically used. The services of the infirmary staff should be available for patients in the hospital beds near their own homes. The local medical profession had an excellent chance to evolve a model co-ordinated hospital service for city and county. The profession should guide the community in hospital matters, and advise the generous how best to meet its hospital needs. Sir Napier Burnett's remarks apply, in principle, to other counties. He has taken a useful step in suggesting that the next move toward a hospital system for each county lies with the medical profession.

### THE CO-ORDINATION MOVEMENT.

AT the recent centenary of the Norfolk and Norwich Eye Infirmary, which is to amalgamate with the Norfolk and Norwich Hospital, Mr. S. H. Burton, representing the medical staff, made some interesting remarks upon the future of hospitals. He was fully in favour of the amalgamation scheme, and foresaw the day when all the principal medical charities would be amalgamated under a central board. The small institutions, he thought would gain thereby in equipment and administration, and the large institutions by the facilities for special treatment for which the small hospitals had often been created. Amalgamation, however, is not exactly a term of precision. The Eye Infirmary at Norwich, for example, is to retain its own identity. Perhaps, therefore, in the larger question to which Mr. Burton turned his attention, co-ordination is the better term. The new Local Committees under the Voluntary Hospitals Commission are a move in this direction, and in our August number we published the resolution of the council of the Prince of Wales' Hospital, Cardiff, in favour of the often-proposed scheme for a central fund for provincial hospitals on the lines of King Edward's Hospital Fund for London. We have always held that the voluntary hospitals can best work out their own salvation, and it is all to the good that such official help as is being given proposes to leave their general independence unimpaired. Now that hospital opinion has come round to recognise the importance of co-ordination in hospital affairs, it is only a question of time before the particular method of achieving it is determined.

### A HOSPITAL'S PROTEST.

COMPLAINTS of hospital patients being sent for lack of a bed to infirmaries are familiar, but it is not often a voluntary hospital board complains that a local authority has "dumped" a patient too ill to be moved upon its wards. The Shrewsbury Hospital has made this complaint against a Montgomeryshire local authority for sending without warning "a dying child" to the institution. It is alleged that there is now a craze among local authorities for the isolation and removal of poor patients, and that orders are often made on the instruction of a sanitary officer, and that once removal has been determined the local authority washes its hands of the affair. The unfortunate child who died soon after arrival at Shrewsbury Hospital is understood to have been in urgent need of an abdominal operation, and the local authority's neglect to warn the hospital or, apparently, to take medical advice in the child's interests, justifies protest and inquiry, even if extenuating facts are brought to light. There is no doubt that the open doors of a voluntary hospital do offer to thoughtless persons, whether local authorities or not, an easy way of shifting responsibility. We are glad that the hospital, in the interests of its patients, has insisted that more consideration shall be shown.

